# Identifying disincentives to ethics consultation requests among physicians, advance practice providers, and nurses: a quality improvement all staff survey at a tertiary academic medical center

**DOI:** 10.1186/s12910-021-00613-7

**Published:** 2021-04-13

**Authors:** Lynette Cederquist, Jamie Nicole LaBuzetta, Edward Cachay, Lawrence Friedman, Cassia Yi, Laura Dibsie, Yiran Zhang

**Affiliations:** 1 Department of Medicine, Division of General Internal Medicine, University of California San Diego, 9500 Gilman Drive #0945, La Jolla, CA 92093-0945 USA; 2 Department of Neurosciences, University of California San Diego, 9300 Campus Point Drive #7740, La Jolla, CA 92037-7740 USA; 3 Department of Medicine, Division of Infectious Diseases, University of California San Diego, 200 W. Arbor Drive #8681, San Diego, CA 92103-8681 USA; 4 Department of Medicine, Division of General Internal Medicine, University of California San Diego, 200 W. Arbor Drive #8985, San Diego, CA 92103-8985 USA; 5 Department of Nursing, University of California San Diego, 200 W. Arbor Drive #8929, San Diego, CA 92103-8929 USA; 6 Department of Family Medicine and Public Health, Division of Biostatistics and Bioinformatics, University of California San Diego, 9500 Gilman Drive, La Jolla, CA 92093 USA

**Keywords:** Ethics consultation, Staff survey, Barriers

## Abstract

**Background:**

Ethics consult services are well established, but often remain underutilized. Our aim was to identify the barriers and perceptions of the Ethics consult service for physicians, advance practice providers (APPs), and nurses at our urban academic medical center which might contribute to underutilization.

**Methods:**

This was a cross-sectional single-health system, anonymous written online survey, which was developed by the UCSD Health Clinical Ethics Committee and distributed by Survey Monkey. We compare responses between physicians, APPs, and nurses using standard parametric and non-parametric statistical methods. Satisfaction with ethics consult and likelihood of calling Ethics service again were assessed using a 0–100 scale using a 5-likert response structured (0 being “not helpful at all” to 100 being “extremely helpful”) and results presented using box plots and interquartile ranges (IQR).

**Results:**

From January to July 2019, approximately 3800 surveys were sent to *all* physicians, APPs and nurses with a return rate of 5.5—10%. Although the majority of respondents had encountered an ethical dilemma (85–92.1%) only approximately half had ever requested an Ethics consult. The primary reason for physicians never having requested a consult was that they never felt the need for help (41%). For APPs the primary reasons were not knowing an Ethics consult service was available (33.3%) or not knowing how to contact Ethics (27.8%). For nurses, it was not knowing how to contact the Ethics consult service (30.8%) or not feeling the need for help (26.2%). The median satisfaction score (IQR) for Ethics consult services rated on a 0–100 scale, from physicians was 76 (29), for AAPs 89 (49), and nurses 70 (40) (*p* = 0.62). The median (IQR) of likelihood of consulting Ethics in the future also on a 0–100 scale was 71 (47) for physicians, 69 (45) for APPs, and 61 (45) for nurses (*p* = 0.79). APP’s and nurses were significantly more likely than physicians to believe that the team did not act on the Ethics consult’s recommendations.

**Conclusions:**

Based on the results presented, we were able to identify actionable steps to better engage healthcare providers—and in particular APPs and nurses—and scale up institutional educational efforts to increase awareness of the role of the Ethics consult service at our institution. Actionable steps included implementing a system of ongoing feedback that is critical for the sustainability of the Ethics service role. We hope this project can serve as a blueprint for other hospital-based Ethics consult services to improve the quality of their programs.

**Supplementary information:**

The online version contains supplementary material available at 10.1186/s12910-021-00613-7.

## Background

Discussion of the function and purpose of ethics consult services has been ongoing in the literature since the 1970s [1–4]. Since that time, ethics consultation has become a codified entity by the American Medical Association [5], are mandated by the Joint Commission for Hospital Accreditation [6] and are endorsed by the Academy for Ethics in Medicine and the American Society for Bioethics and Humanities. While not the initial purpose of ethics services, consultation in some studies has been shown to improve certain outcomes such as decreased length of stay and provider and patient/family satisfaction [7]. Despite being well-established entities in hospitals throughout much of the world, the availability of and qualifications among ethics services appear to vary between—and even within—institutions [7–10]. Healthcare providers have heterogenous perceptions of ethics services’ effectiveness that impact its utilization [11–15]. Quality improvement efforts have been conducted at institutions in part to address the variations in quality [16, 17]. Most recently, the American Society of Bioethics and Humanities has implemented the Healthcare Ethics Consultant-Certified Program in order to establish a national standard in the United States for the practice of clinical healthcare ethics consulting.

Several reasons influence an individual’s propensity for not calling an ethics consult even though an ethical dilemma is present. Reasons such as perceived delays in clinical decision-making, lack of confidence in the qualifications of the consultants, lack of familiarity with the process, desire not to involve more people in the care of the patient, and a sense that one should be able to manage patient issues oneself [11, 13, 18]. Prior studies have focused specifically on clinician satisfaction and barriers to ethics consultation [11, 12]. However, there is scarce information on our understanding of the disincentives why clinicians including physicians, advance practice providers (APPs), and nurses do not call an ethics consult after contemplating this possibility or the potential differences in disincentives between these three provider types. [11–13, 17].

Similar to other institutions [19, 20], we conducted this survey-based quality improvement study to better understand the reasons for, perception of, and limitations to ethics consultation in a large academic urban tertiary referral center. Our Ethics consultation service is comprised of five consultants; an MD (medical doctor), a DO (doctor of osteopathy), a physician assistant, and two registered nurses who rotate calls weekly. On average, our service conducts 115 consults annually. Our consult service encompasses three hospitals and a cardiovascular center with a total of 808 beds. Our consult service is available 7 days a week, 24 h a day.

Cognizant of the inherent response-bias effect of any survey methodology, we focused on three main study aims: to 1) understand some of the reasons why physicians, APPs, and nurses had never requested an ethics consult, 2) understand the reasons providers who called an ethical consult previously would not consider calling one again, and 3) evaluate whether there is a significant difference in the reasons for which doctors, APPs, and nurses do not request an ethics consultation.

## Methods

### Study design

This was a cross-sectional survey-based study within a single health system.

### Survey conception

Members of the ethics committee devised an internal survey tool (see Additional file 1) to assess respondents’ awareness of, previous experiences with, and perceptions regarding the Ethics consultation service. The questionnaire inquired first about the respondent’s highest degree, specialty, service/location within the organization, whether they worked in an inpatient or outpatient setting, and length of employment at our institution. The second page contained the question “Have you ever encountered an ethical dilemma in the course of caring for a patient?” to gauge internal validity, as the vast majority of individuals have encountered an ethical dilemma during their medical profession. We then asked respondents to indicate whether they had ever called an ethics consult and if so, to indicate the reasons for requesting a consult. If they responded that they had never requested a consult, we asked them to identify reasons they had not done so. For those who had requested consults, we asked whether the consult and recommendations were helpful using a 0–100 scale using a Likert-like structured response (0 being “not helpful at all” to 100 being “extremely helpful”). We also included specific logistical questions, such as (1) Was the consult completed in a timely manner; (2) Do you believe the treating team acted on the Ethics service consultant’s recommendations? Respondents were asked to indicate the likelihood that they would call for an ethics consult in the future (again, using a Likert-like response structure codified in a scale of 0–100 scale). Respondents who indicated they would be unlikely to request future consults were asked to provide reasons via comments.

### Participants and recruitment

In January 2019, we sent out an internally devised survey (Additional file 1) to 1517 physicians and 277 APPs to seek feedback regarding the use of our ethics consultation service. The same survey was sent to approximately 2000 nursing staff in July of 2019. We chose the SurveyMonkey platform to optimize the user interface on mobile devices. This was considered a quality improvement (QI) project, which was exempt from IRB approval. As an incentive to complete the survey, we offered a raffle to win one of three $100 Amazon Gift cards, though participation was not required in order to enter the raffle.

### Data analysis

Descriptive statistics are presented using means with standard deviations and for non-normally distributed data, variables such as medians (with interquartile ranges [IQR]), frequencies, and percentages) were used. To compare responses between physicians, APPs, and nurses, we used χ^2^ test or Fisher’s exact test (when the expected values in one of the cells of the contingency table < 5) for comparison of categorical variables and 2-sided *t*-test (or Kruskal–Wallis Rank Sum Test) for numerical variables. We created box plots to depict the distribution of responses for satisfaction with ethics consult and the likelihood of calling the Ethics consult service again.

## Results

We received responses from 150 out of 1517physicians (10% response rate), 35 out of 277 APP’s (11.5% response rate), and 109 out of 2000 nurses (5.5% response rate) for a total of 295 responses. The physicians and APPs were from an array of specialties and subspecialties including: hospitalist/internal medicine, surgery (colorectal, general, cardiothoracic, neurosurgery), family medicine, emergency medicine, psychiatry, critical care (pulmonary critical care, anesthesia critical care, neurocritical care), anesthesiology/pain, infectious diseases/HIV, pathology/neuropathology, Obstetrics/Gynecology, and seventeen other subspecialties. The majority (80%) of respondents had been employed at UCSD Health for greater than 5 years. The majority of respondents worked in the inpatient settings, either full or partial time (82.4%). The majority of respondents (92% physicians, 88% APPs, and 85% nurses) indicated that they had encountered an ethical dilemma at some point. Nurses reported ever requested an ethics consult at a lower rate (35%) compared to physicians (51%) and APPs (63%) p-value 0.029. (Table 1). Specific reasons identified for having requested a consult varied between disciplines as described in Table 2.

**Table 1:** Survey results

	Physicians	APPs	Nursing	*p *Value
Employed at our institution ≥ 5 years	*n* = 150	*n* = 35	*n* = 109	
Less than 5 years	28 (18.7%)	1 (2.9%)	15 (13.8%)	0.056
More than 5 years	122 (81.3%)	34 (97.1%)	94 (86.2%)	
Inpatient/Outpatient	*n* = 150	*n* = 35	*n* = 106	
Both	91 (60.7%)	13 (37.1%)	0 (0.0%)	< 0.001
Inpatient	27 (18.0%)	11 (31.4%)	101 (95.3%)	
Outpatient	32 (21.3%)	11 (31.4%)	5 (4.7%)	
Encountered an ethical dilemma	*n* = 151	*n* = 33	*n* = 107	
No	12 (7.9%)	4 (12.1%)	16 (15.0%)	0.203
Yes	139 (92.1%)	29 (87.9%)	91 (85.0%)	
Requested an ethics consult	*n* = 150	*n* = 33	*n* = 107	
No	73 (48.7%)	18 (54.5%)	70 (65.4%)	0.029
Yes	77 (51.3%)	15 (45.5%)	37 (34.6%)	
Likelihood of consulting Ethics in the future	*n* = 145	*n* = 29	*n* = 97	
0–100 scale: mean (standard deviation)	66.99 (29.76)	64.17 (29.82)	65.64 (27.34)	0.867
Rate the effectiveness of the ethics consult participation and recommendations	*n* = 83	*n* = 13	*n* = 43	
0–100 scale: mean (standard deviation)	71.60 (23.76)	68.46 (36.58)	66.77 (27.32)	0.608
Consult completed in timely manner	*n* = 81	*n* = 14	*n* = 41	0.314
No	6 (7.4%)	4 (28.6%)	11 (26.8%)	
Yes	75 (92.6%)	10 (71.4%)	30 (73.2%)	
Do you believe the team acted on the ethics recommendations?	*n* = 81	*n* = 14	*n* = 41	0.007
No	6 (7.4%)	4 (28.6%)	11 (26.8%)	
Yes	75 (92.6%)	10 (71.4%)	30 (73.2%)

**Table 2:** Reasons for requesting a consult

	Physician (*n* = 83)	NPPs (*n* = 14)	Nurses (*n* = 43)	*p* Value
Assistance with treating an unrepresented patient	37 (44.6%)	7 (50.0%)	11 (25.6%)	0.081
Mediate conflict	26 (31.3%)	3 (21.4%)	20 (46.5%)	0.127
Limitation or withdrawal of treatment or change of code status	57 (68.7%)	9 (64.3%)	22 (51.2%)	0.155
Clarify the appropriate surrogate	16 (19.3%)	4 (28.6%)	18 (41.9%)	0.025
Address uncertainty regarding the patients decision making capacity	36 (43.4%)	5 (35.7%)	13 (30.2%)	0.347
Others	7 (8.4%)	2 (14.3%)	4 (9.3%)	0.748

The primary reason for physicians never having requested a consult was that they never felt the need for help (41%). For APPs, the primary reasons were not knowing an Ethics consult service was available (33.3%) or not knowing how to contact Ethics (27.8%). For nurses, top reasons included not knowing how to contact the Ethics consult service (30.8%) or not feeling the need for help (26.2%) (Table 3). The most striking difference between the three groups was their responses to “Did not feel the attending of record would agree”; only 2.7% of physicians included this reason, while 16.7% of APPs and 16.9% nurses selected this reason. 58% of respondents selected “Other” which were at least partially captured by comments entered by the respondents (Table 3). Some of the more common “other” reasons for having never requested an Ethics consult among physicians and APPs were:

- “I was not the attending of record.”
- “I was a consultant.”
- “I consulted Risk Management instead.”

**Table 3:** Reasons for having never requested an Ethics consult

	Physicians *(n* = 73)	APPs (*n* = 18)	Nurses (*n* = 65)	*p *Value
Did not know there was an ethics consult service	24 (32.9%)	6 (33.3%)	16 (24.6%)	0.529
Did not know how to contact the ethics consult service	12 (16.4%)	5 (27.8%)	20 (30.8%)	0.129
Never felt the need for their help	30 (41.1%)	4 (22.2%)	17 (26.2%)	0.127
Did not believe ethics consults are helpful	0 (0.0%)	0 (0.0%)	4 (6.2%)	0.064
Slow down the decisions needed to be made or further complicate the situation	7 (9.6%)	1 (5.6%)	5 (7.7%)	0.919
Did not feel the attending of record would agree	2 (2.7%)	3 (16.7%)	11 (16.9%)	0.008
Other	48 (65.8%)	9 (50.0%)	34 (52.3%)	0.208

Nurse responses under “other” included a different focus:

- “Out of my scope of practice”.
- “Ethics is powerless in the face of powerful surgeons”.
- “I followed the chain of command”
- “Did not know nursing could call Ethics”.
- “I’ve seen a consult but did not improve the situation.”

**Fig. 1:**
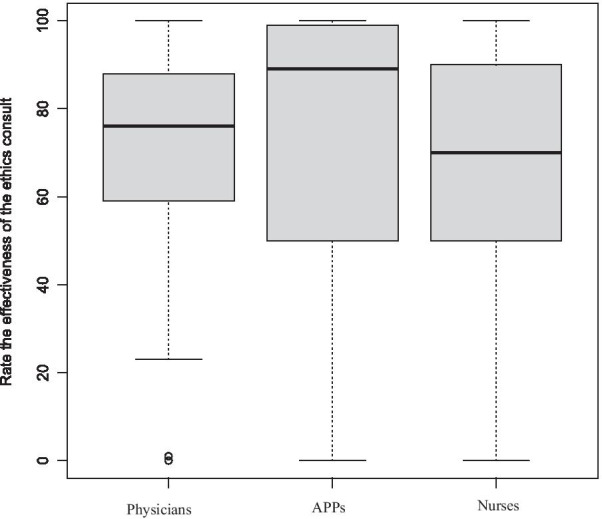
Boxplot depicting rating score distribution of the effectiveness of the ethics consult, 0–100

**Fig. 2:**
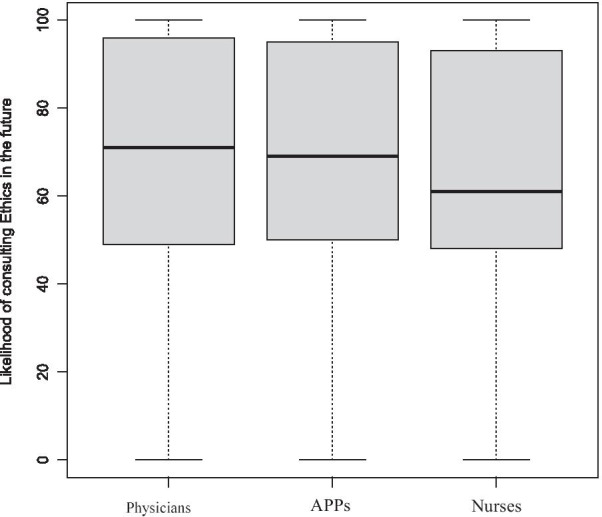
Boxplot reflecting response score distribution of the likelihood of consulting ethics in the future

Of those who had previously requested an ethics consultation, the reasons for requesting a consult included assistance with treating an unrepresented patient (patients who lack decision making capacity and have no surrogate), mediating conflict, limitation of life-sustaining treatment, clarification of appropriate surrogate, and uncertainty regarding the patient’s decision-making capacity (Table 2). When asked to rate the effectiveness of the Ethics consult on a scale 0–100, the median response and interquartile range (IQR) was 71 (47) for physicians, 69 (45) for APPs, and 61 (45) for nurses. (Table 4). Although the nurses were less satisfied with the consult service’s effectiveness, there was overlap, as illustrated in boxplot (Fig. 1). For responses to “likelihood of requesting a consult in the future” on a 0–100 continuous scale, the calculated median (IQR) scores were 71 (47) for physicians, 69 (45) for APPs, and 61 (45) for nurses, see Table 4. There was once again overlap between the three disciplines as illustrated in Fig. 2. Of those respondents who were unwilling to call an ethics consultation in the future, general reasons given included: 1) prior experience with a consult was poor, 2) disagreement with recommendations, 3) variability in the quality of the Ethics consultants/recommendations, and 4) lack of specific recommendations from the Ethics consultant. Responses to this question were solicited solely by requesting comments, not selected from a pre-defined list of choices. We therefore could not quantitate the reasons provided. Some specific comments from physicians and APPs included:

- “Prior experience with ethics consult would make it much less likely that I would ever request such a consult.”
- “I disagreed with Ethics’ recommendations which seemed just plain wrong.”
- “Some Ethics consultants are much more helpful than others.”
- “We will exhaust all other options before going to Ethics.”
- “More physical presence would be nice to discuss recommendations in depth.”
- “The incident I am thinking of had to do with ‘yielding’ to the wishes of the family, which could have been detrimental or fatal. I believe was just plain wrong under the circumstances.”
- “In some cases, it has been 2–3 days before the consultant documents, and the documentation is very general or brief.”

**Table 4:** Median and interquartile range (IQR) of likelihood of consulting ethics and rating of the effectiveness of the ethics consult on a 0–100 scale between physicians, APPs, and Nurses: p-value was calculated by performing Kruskal–Wallis Rank Sum Test

	Physicians	APPs	Nurses	*p *Value
Likelihood of consulting Ethics in the future	71 (47)	69 (45)	61 (45)	0.79
Rate the effectiveness of the ethics consult	76 (29)	89 (49)	70 (40)	0.615

Some of the comments from nurses included:

- “Ethics just rubberstamps for the doctors.”
- “Even with patients who have had ethics consults, unethical decisions are made.”
- “Ethics does not want to tell the medical team how to care for their patients.”

Lastly, 92.6% of physicians believed the team had acted on the Ethics consultants’ recommendations, but only 71.4% of APPs and 73.2% of nurses agreed with this statement (Table 2).

## Discussion

In conclusion, we found a diversity in reasons for not calling ethics consults, but for APPs and nursing, these reasons included the practicality of not realizing the service was available or that they could call a consult themselves—the *how to*. In contrast, physician experience was less focused on the practicality of *how* to interact with the Ethics service, and more likely to focus on the *why* (e.g. not felt to be needed, disagreed with recommendations). In this way, APPs experience with Ethics consult services aligns more with nursing staff than with physicians; additionally, nurses and APPs were both more likely to list “attending would not agree” as their reason for not requesting a consult, suggesting a hierarchical nature. At our institution, we found four general reasons people did not consult Ethics: (1) unawareness of the existence of or means of contacting the Ethics service; (2) perceptions that an Ethics consult would not be helpful or might slow down decision making; (3) having experienced a poor quality consult in the past, including variability in knowledge and ability among various consultants; (4) a lack of specific guidance from the consultant.

### Participants who had never consulted ethics

The proportion of responses indicating no awareness of the existence of our Ethics consultation service or how to contact us which was unexpected and contrary to our perception that we are well known and easy to contact with a consultant available 24/7 who can be paged by anybody involved in the care of the patient. This was also surprising because the majority of respondents had worked at our institution for over 5 years. As such, it was a call for us to scale up awareness of our services using different within-institution campaigns.

The other reasons individuals had never requested a consult are similar to other studies previously published [12, 19, 20]. Some of these perceptions may reflect some of the reality of the ethics consultation process, which does need to allow time for a more deliberative process that may require extra time and therefore slow down decision making with the goal of achieving higher quality decision making.

There was a significant difference between physicians and APPs/nurses who identified “did not feel the attending physician would agree with an Ethics consult” as a reason for not requesting a consult. Ethics consult services are unique from other consult services by virtue of the fact that consult requests can be initiated by other members of the team with or without the consent of the attending physician. Despite that access, other team members felt that they should not request a consult without the permission of the attending, and this represents a significant barrier for them. Further, feeling as though you cannot call an ethics consult because the attending would/does not agree—even if you perceive an ethical dilemma—may contribute to burnout [21].

Many individuals indicated that when faced with an ethical dilemma, they did not feel they needed help resolving the dilemma (41.1% of physicians, 22.2% of APPs, and 26.2% of nurses). This likely reflects the assumption that every clinician demonstrates ethically sound decision-making skills without the need for an ethics consult to resolve every ethical dilemma encountered. However, the remaining uncertainty is whether some of these situations might have benefitted from the involvement of an ethics consult with clinicians not recognizing the need for help.

### Participants who had consulted ethics, but would not re-consult

The reasons individuals who had consulted Ethics in the past gave for not planning to re-consult Ethics in the future provide insight into into additional action areas for our consult service. These deficiencies included: variability in expertise among consultants, absence of specific guidance, and lack of the consultant's physical presence. In comparing nursing responses to physicians and APPs, nurses more frequently expressed a perception of being less able to impact the course of care for a patient. This was reflected in statements such as “Ethics does not want to tell the medical team how to care for their patients”, “waste of time”, “Ethics just rubberstamps decisions made by surgeons”, and “even with patients who had an Ethics consult, unethical decisions were made.” These comments convey the nurses’ hopes that an Ethics consult would effect a change in patient care, and frustration when it did not. This sense of powerlessness can often contribute to moral distress and burnout among nursing [21, 22]. Also notable was the lower percentage of nurses (73.2%) and APPs (71.4%) compared to physicians (92.6%) who believed the team had acted on the Ethics consultant’s recommendations. This reflects the reality that Ethics recommendations are purely advisory. The attending physician is ultimately the person who determines the course of action which will be taken. If he/she did not initiate the Ethics consult request, there is a lower likelihood they will act on the recommendations. Clarification of our role when a consult is requested may help to gauge expectations for all team members.

### Limitations

Inference from the study is subject to several limitations. First, we had a low rate of responses, just 10% for physicians, 11.5% for APPs, and 5.5% for nursing. Some of the reasons for this include that we could not limit the survey distribution to only inpatient clinicians, we were only able to send the survey out one time to each group, and we were not able to publicize the survey before sending it out. We chose to send out a one time all-staff survey to achieve the broadest sampling rather than a more targeted survey, which would have likely achieved a higher response rate. In addition, for those who did respond, many of them did not respond to all of the questions, so some questions had an even lower rate of response. Despite these limitations, the respondents' absolute numbers were higher than many similar surveys in the literature and included a broad sampling of specialties and subspecialties. Future survey studies could be done with a more focused sampling of particular services or disciplines.

A second limitation was the limited data collected regards the reasons respondents would not re-consult Ethics. We utilized only comments that respondents could complete, rather than a pre-defined list of possible options. Consequently, only a limited number of respondents took the time to enter a comment explaining their reasons for not re-consulting. Thus, our results likely underrepresent the reasons for not calling an Ethics consult.

Finally, our study findings may not be generalizable to other populations with different cultural perceptions or across different types of health system systems or access to health systems.

## Conclusions

Despite limitations, our study identified important actionable steps that we will implement with the plan to reassess their impact on our Ethics consult services with the goal of increased utilization of our service. Since the completion of this survey, we have taken specific steps in order to accomplish these goals including:

1. More actively engage with nursing and APPs, including education and regular ethics rounds.
2. Disseminate information regarding the availability of our consult service more prominently, including a dedicated site on our hospital’s intranet and information regarding *who* can place such consults (i.e. everyone).
3. Provide ongoing education of consultants to provide specific recommendations and guidance, including regular reviews of our consult notes using the EQUAT tool [23].
4. Implement use of feedback surveys of staff following each Ethics consult to solicit real time feedback.
5. Conduct biannual staff training in clinical ethics seminars.
6. Conduct weekly Ethics rounds in three of our critical care units.

More importantly, we hope to provide an actionable model that could be replicated by others adjusted to their health system or culture practice.

## Supplementary information

**Additional file 1**. Survey Tool.

## Data Availability

The datasets used and/or analyzed during the current study are available from the corresponding author on reasonable request.

## Abbreviations

APP: Advance practice providers which includes nurse practitioners (NP) and physician assistants (PA)
MD: Medical doctor
DO: Doctor of osteopathy
RN: Registered nurse
BSN: Bachelors of science in nursing
IRB: Institutional review board
QI: Quality improvement
