# Supplementary material for: Identifying disincentives to ethics consultation requests among physicians, advance practice providers, and nurses: a quality improvement all staff survey at a tertiary academic medical center
Source: BMC Med Ethics. 2021 Apr 13;22:44. doi: 10.1186/s12910-021-00613-7 (PMC8045298; doi:10.1186/s12910-021-00613-7)
Supplement: Supplementary file 1 — Additional file 1. Survey Tool. [file 12910_2021_613_MOESM1_ESM.docx]

**Appendix 1: Survey Tool**

1. By participating in this survey, you are not guaranteed to win a prize; however, you do not need to participate in the survey to have a chance of winning a prize. Will you participate in our survey?

Yes

No

2. Please indicate your healthcare role:

MD/DO

NP/PA

RN

3. MD/DO:

Resident

Fellow

Attending

4. How long have you been an attending MD/DO? NP/PA? RN/BSN?

< 5 years

5-10 years

> 10 years

5. What is your specialty?

6. Do you currently work in the inpatient or outpatient setting?

Inpatient

Outpatient

Both

7. Have you ever encountered an ethical dilemma in the course of caring for a patient?
Yes

No

8. Have you ever requested an Ethics Consult while training or employed at UCSD?

Yes

No

9. If yes, how many consults have you requested in the past 2 years?

10. What kind of help were you hoping for? (choose all that apply)

Assistance with treating an unrepresented patient

Clarify the appropriate surrogate

Limitation/withdrawal of treatment or change of code status

Mediate conflict

Address uncertainty regarding the patient’s decision-making capacity

Other:

11. How would you rate the Ethics consultation participation and recommendations?

0 – 100 scale from “not helpful at all” to “somewhat helpful” to “extremely helpful”

12. Was the consultation completed in a timely manner?

Yes

No

13. Do you believe the treating team acted on the Ethics consultant’s recommendations?
Yes

No

14. What is the likelihood that you will call for an ethics consult in the future?

0-100 scale from “definitely not” to “somewhat likely” to “extremely likely”

15. Any additional comments/feedback:
